# Adipocyte dysfunction promotes lung inflammation and aberrant repair: a potential target of COPD

**DOI:** 10.3389/fendo.2023.1204744

**Published:** 2023-10-10

**Authors:** Si-jin Zhang, Xian-zheng Qin, Jie Zhou, Bin-feng He, Surendra Shrestha, Jing Zhang, Wei-ping Hu

**Affiliations:** ^1^ Department of Pulmonary and Critical Care Medicine, Ruijin Hospital, Shanghai Jiao Tong University School of Medicine, Shanghai, China; ^2^ Department of Gastroenterology, Ruijin Hospital, Shanghai Jiao Tong University School of Medicine, Shanghai, China; ^3^ Department of Hematology, Tongji Hospital of Tongji University, Tongji University School of Medicine, Tongji University, Shanghai, China; ^4^ Department of Pulmonary and Critical Care Medicine, Zhongshan Hospital, Fudan University, Shanghai, China; ^5^ Emergency Department, Om Aasha Hospital Pvt. Ltd., Dhanghadi, Nepal

**Keywords:** adipocyte dysfunction, Chronic Obstructive Pulmonary Disease, lung inflammation, aberrant repair, adipocytokines

## Abstract

**Background:**

Obesity and chronic obstructive pulmonary disease (COPD) are prevailing worldwide, bringing a heavy medical burden. Clinical and pathophysiological relationship between obesity and COPD is paradoxical and elusive. We aim to explore their inherent associations from clinical, genetic, and animal levels.

**Methods:**

We performed literature review and cohort analysis of patients with COPD to compare lung function, symptom, and prognosis among different weight groups. After retrieving datasets of obesity and COPD in Gene Expression Omnibus (GEO) database, we carried out differentially expressed gene analysis, functional enrichment, protein–protein interactions network, and weighted gene co-expression network analysis. Then, we acquired paraffin-embedded lung tissues of fatty acid–binding protein 4–Cre-BMPR2^fl/fl^ conditional knockout (CKO) mice that were characterized by adipocyte-specific knockout of bone morphogenetic protein receptor 2 (BMPR2) for staining and analysis.

**Results:**

Our cohort study reports the effect of obesity on COPD is inconsistent with previous clinical studies. Lung function of overweight group was statistically superior to that of other groups. We also found that the inflammatory factors were significantly increased hub genes, and cytokine-associated pathways were enriched in white adipose tissue of patients with obesity. Similarly, injury repair–associated genes and pathways were further enhanced in the small airways of patients with COPD. CKO mice spontaneously developed lung injury, emphysema, and pulmonary vascular remodeling, along with increased infiltration of macrophages. BMPR2-defiecient adipocytes had dysregulated expression of adipocytokines.

**Conclusion:**

Inflammation and abnormal repair might be potential mechanisms of the pathological association between obesity and COPD. BMPR2-associated adipocyte dysfunction promoted lung inflammation and aberrant repair, in which adipocytokines might play a role and thus could be a promising therapeutic target.

## Introduction

Characterized by chronic inflammation, remodeling of small airways, and emphysema (1), chronic obstructive pulmonary disease (COPD) has been a major public health problem. More than 380 million people are estimated to suffer from COPD worldwide (2). World Health Organization predicted that COPD would be the third leading cause of death and rank the fifth in the burden of disease (1). Poor prophylaxis and management of COPD also increase patient’s morbidity and mortality (3, 4).Thus, it is important to understand pathogenesis of COPD, which could guide us for better management of COPD.

With lifestyle changes, more people became overweight and obese rather than underweight. The 2014 Global Non-communicable Disease Survey showed that about 40% of adults were overweight and 10.8%–14.9% were obese (5). Body mass index (BMI) had been widely used to access prevalence of obesity. It has been well established that increasing BMI is a major risk factor for cardiovascular disease (6), osteoarthritis (7), diabetes mellitus (8), and choledocholithiasis (9). However, the relationship between obesity and clinical outcome of COPD seems to be more complex (10). Some studies report that both adiposity and underweight were risk factors for COPD (11). In contrast, other studies found overweight to significantly improved the survival of patients with COPD, which was defined as “obesity paradox” (12). From the perspective of respiratory physiology, patients with obesity with COPD may experience lower lung hyperinflation because of modified mechanical properties of chest wall, compared with their lean counterparts (13). Moreover, patients with overweight with COPD often present a mild degree of emphysema, probably accounting for decreased mortality (14).

It was hard to elucidate the sophisticated effects of the adipose tissue on lung (13). Regulation of adipose tissue function could be a promising therapeutic target, because it acts as a systemic regulator in response to the environmental changes, like inflammation (15). Similar to COPD, increased systemic inflammation was also associated with excessive fat mass, especially regarding tumor necrosis factor–α (TNFα), interleukin-6, C-reactive protein, and metabolic syndrome (12). There have been several classical meta-analyses on the relationship between BMI and prognosis or mortality of patients with COPD. For example, Evangelos et al. reported that BMI was one of the most commonly used index in the development of prognostic models of COPD (16). Xiong et al. reported that patients with COPD being overweight or obese had a protective effect against mortality (17). The phenomenon of “obesity paradox” also exists in COPD animal models. For cigarette smoke-exposed rodents, the obese mice presented more severe emphysema and pulmonary inflammation compared with lean group (18). For rodents who received lipopolysaccharide of *E. coli*, obese rats showed less deterioration of lung function, lower phagocytosis of monocytes in blood and macrophages in adipose tissue, as well as reduced migration of neutrophils in comparison with normal weight rats (19). Moreover, Wu et al. reported that obesity could alleviate ventilator-induced lung injury through Signal transducer and activator of transcription 3-Suppressor of cytokine signaling 3 (STAT3–SOCS3) signaling pathway (20).

Adipocytokines are a class of bioactive mediators secreted by adipocytes, including leptin, adiponectin, fibroblast growth factor 21, bone morphogenetic protein–4 (BMP4), and BMP7, with ability of regulating systemic inflammation and remodeling (21, 22). BMP4 and BMP7 act their function by binding onto the receptors including bone morphogenetic protein receptor 2 (BMPR2). BMPR2-deficient adipocytes were susceptible to pyroptosis and apoptosis (23). Qian et al. observed that the fatty acid–binding protein 4 (FABP4)–Cre-BMPR2^fl/fl^ mice, with the adipocyte-specific knockout of BMPR2, begin to weaken and die after 3 weeks of weaning, with an adult survival rate of only 25% (23). However, it is not clear whether adipose tissue dysfunction in FABP4-Cre-BMPR2^fl/fl^ mice impacts the lungs. Our study aims to investigate the effect of adipocyte dysfunction on lung inflammation and lung injury and hopes to develop a better understanding of the molecular targets for COPD therapy.

We reviewed the clinical studies of “COPD” and “obesity.” Then, we summarized their clinical characteristics and relationships with obesity from the cohort of our center. We downloaded raw data for gene expression profile from GEO database, which included subcutaneous adipose tissues (GSE11906) (24) and small airways (GSE151839) (25). The differentially expressed genes (DEGs) were obtained and were further subjected to gene set enrichment analysis (GSEA), Kyoto Encyclopedia of Genes and Genomes (KEGG), and protein–protein interaction (PPI) network analysis. At the same time, weighted gene co-expression network analysis (WGCNA) was conducted to explore the specific key modules. Finally, we validated lung injury and emphysema in the lungs of FABP4-Cre-BMPR2^fl/fl^ mice by hematoxylin–eosin (H&E) stain and immunostaining, revealing partial results consistent with public database analysis. Re-analysis of the RNA sequencing and prediction of the related genes between FABP4 and BMPR2 were assessed to illustrate the role of adipocytokines.

## Methods

### Literature review

To investigate the clinical relationship between COPD and obesity, we searched PubMed (http://www.ncbi.nlm.nih.gov/pubmed) for relevant studies (March 2013 to March 2023), using keywords “COPD” and “obesity.” The following critical elements were extracted and recorded, including study population, obesity-related definition, with or without interference, outcome, and statistical method.

### Patient cohort

To compare the difference among patients with COPD with different BMI, we re-analyzed the clinical data within a prospective cohort. Detailed description of this cohort has been published in our previous studies (26, 27). In brief, we recruited patients hospitalized for AECOPD (acute exacerbation of COPD) in the Department of Pulmonary Medicine of Shanghai Zhongshan Hospital between January 2015 and July 2017, collected their clinical information, and followed up their status about recurrent AE (re-AE) and survival within 1 year after discharge. Inclusion criterion was that patients with a clearly recorded COPD history had deteriorated respiratory symptoms, which was categorized as AECOPD by two separate pulmonologists. Exclusion criteria were exacerbations induced by other respiratory diseases, such as asthma, bronchiectasis, congestive heart failure, pleuritis, pneumothorax, pulmonary embolism, and restrictive lung disease. Clinical outcomes were days of in-hospital stay, hospitalized death, and re-AE within 1, 3, 6, and 12 months. Re-AE was defined as the new worsening of respiratory symptoms lasting for over 2 days, which required additional medical intervention. The prospective cohort was approved by Institutional Review Board (IRB) of Shanghai Zhongshan Hospital (Certificate No. B2015-119R).

### Gene expression data and differential analysis

We identified the shared DEGs of COPD and obesity. Gene expression datasets of subcutaneous adipose tissues (GSE11906) (24) and small airways (GSE151839) (25) were retrieved from publicly available GEO database. Obesity-associated differential gene analysis was performed in white adipose tissues from between overweight volunteers (n = 10) and normal weight volunteers (n = 10). COPD-associated DEGs were analyzed between non-smokers (n = 24) and patients with COPD (n = 36) by performing the R package “limma.” Genes with an adjusted *P*-value <0.05 and log2 fold change >1.2 were considered as being differentially expressed. Volcano plot was performed using the R package ggplot2 version 3.2.1 to visualize the DEGs. R package “venneuler” was used to draw a Venn diagram. The shared DEGs were retained for further analysis.

### Gene set enrichment as well as Kyoto Encyclopedia of Genes and Genomes analyses

GSEA of the DEGs was conducted to investigate the biological characteristics of COPD and obesity. False discovery rate (FDR) <0.25, Nominal (NOM) p-value <0.05, and Normalized enrichment score (NES) >1 were considered significant enrichment. Furthermore, we used R package “clusterProfiler” to conduct KEGG analyses of the DEGs. P < 0.05 was considered statistically significant. We used the R software “ggplot2” to visualize above results.

### Protein–protein interaction network construction and hub gene selection

To identify hub regulatory genes and to examine the interactions between DEGs, PPI network was generated with an open-source software Cytoscape (version 3.9.1), which is based on the results of Search Tool for the Retrieval of Interacting Genes/Proteins (STRING; https://string-db.org/) (28). These interactions among proteins with a combined score >0.4 were considered statistically significant. Furthermore, hub genes were selected with Molecular Complex Detection (MCODE), which is a free plugin in Cytoscape. In addition, the parameters were set as degree cutoff = 2, haircut on, node score cutoff = 0.2, k-core = 2, and maximum depth = 100.

### Weighted gene co-expression network analysis

We preformed WGCNA to find the key modules. We used the “WGCNA” package in R to build a co-expression network targeting DEGs to construct specific modules and analyze the module–phenotype relationship as previously described (29). Subsequently, the adjacency matrix was transformed into a topological overlap matrix (TOM), and average linkage hierarchical clustering was used to construct a clustering dendrogram of the TOM matrix. Then, gene hierarchical clustering dendrogram was made to identify co-expression modules, and the module eigengene (ME) was calculated. The minimal gene module size was set to 30 to obtain appropriate modules, and the threshold to merge similar modules was set to 0.25. Finally, we predicted correlation between ME and phenotype to reveal the clinical traits.

### Hematoxylin–eosin stain of lung tissues

FABP4 is an adipocyte-specific promoter. FABP4-Cre-BMPR2^fl/fl^ (CKO) mice are characterized by adipocyte-specific knockout of BMPR2. As Qian et al. reported (23), mice bearing both FABP4-cre and BMPR2 flox/flox genotypes were first crossed to breed FABP4-Cre-BMPR2^fl/−^ (CKO heterozygote) mice. Then, CKO heterozygote mice were further crossed to breed FABP4-Cre-BMPR2^fl/fl^ (CKO homozygote) mice, which were identified by PCR and DNA electrophoresis. Primers of FABP4-Cre are GGTCGATGCAACGAG TGATGAGGT and CAGCATTGCTGTCACTTGGTCGTG. Primers of BMPR2-flox are TTATTGTAAGTACACTGTTGCT and GGCAGACTCTGACTTTGACGC. Qian et al. previously isolated cells from inguinal white adipose tissue of wild-type (WT) and CKO mice and performed Western blots to successfully confirm decreased levels of BMPR2 in CKO mice’s adipose tissue (23, 30).

Qian et al. kindly granted us three paraffin-embedded lung tissues, including one WT and two CKO mice, all of which were sacrificed and collected on days 19–22. We made serial sections of these tissues and performed H&E staining and digital photographic scan on every eight slides. CaseViewer software (3D HISTECH Ltd., Hungary) was used to measure the indices of H&E stain. Lung injury score was divided into five grades: normal as grade 1, focal interstitial congestion as grade 2, diffuse congestion as grade 3, focal consolidation as grade 4, and diffuse consolidation as grade 5 (at ×5 magnification).

### Immunostaining

Detailed protocols of immunohistochemical stain and immunofluorescence stain were reported in our previous study (31). We used the Super Plus™ High Sensitive and Rapid Immunohistochemical Kit (E-IR-R221, Elabscience^®^, China) and the Immunofluorescence Application Solutions Kit (12727, Cell Signaling Technology (CST), USA). The list of antibodies is shown in **Supplementary Table 1**.

### Terminal deoxynucleotidyl transferase–mediated Deoxyuridine Triphosphate-biotin nick end labeling assay

The one-step terminal deoxynucleotidyl transferase (TdT)–mediated Deoxyuridine Triphosphate (dUTP)-biotin nick end labeling (TUNEL) *In Situ* Apoptosis Kit (E-CK-A321, Elabscience^®^, China) was used to detect apoptotic cells in lung tissues. After dewaxing and hydrating, the paraffin sections were incubated with Proteinase K for permeation. Positive control was extra incubated with Deoxyribonuclease I (DNase I) buffer. Working solution was prepared by mixing TdT equilibration buffer, labeling solution, and TdT enzyme and then added onto the paraffin sections for 1 h. After sufficient washing and nuclear staining, we sealed the paraffin sections and recorded photographs using fluorescence microscope.

### Search the related genes of Bone Morphogenetic Protein Receptor Type 2 and Fatty Acid Binding Protein 4

The single protein name (“Bone Morphogenetic Protein Receptor Type 2” or “Fatty Acid Binding Protein 4”) and organism (“Homo sapiens”) were searched at STRING website. The main parameters were set as minimum required interaction score [“low confidence (0.150)”], meaning of network edges (“evidence”), maximum number of interactors to show (“no more than 50 interactors” in the first shell), and active interaction sources (“experiments”). Finally, we obtained the available experimentally determined BMPR2-binding proteins and FABP4-binding proteins.

### Statistical analysis

Categorical variables were showed as number (percentage) and compared by the Chi-square test. Continuous variables were expressed as the mean and standard deviation, which were analyzed by Kruskal–Wallis test for three different groups or Mann–Whitney test for two groups. We used GraphPad Prism 6 (GraphPad Software, CA, USA) and IBM SPSS statistics 23 (SPSS Inc., Chicago, IL, USA) to perform the above statistical analysis and defined the two-tailed *P* < 0.05 as statistical significance.

## Result

### Effect of obesity on COPD is inconsistent across various clinical studies

In **Table 1**, 15 studies on COPD and obesity are listed. Four studies reported that overweight or obesity was beneficial to the survival for patients with COPD, and three studies showed that patients with overweight or obesity had a decreased risk of acute exacerbation. However, three other studies reported that obesity could impair exercise capacity of patients with COPD. Some large-scale cross-sectional studies demonstrated that people with overweight or obesity had increased incidence of COPD, which might be associated with aggravated response to cigarette smoke or pollutants. Thus, the effect of overweight or obesity on COPD is inconsistent among current studies.

**Table 1:** A literature review of clinical studies on COPD and obesity.

PMID	Country	Population		Definition	Outcome	
32586139	USA	AECOPD	301	BMI > 30	Mortality at 6 month and 1 year after AE	Improve
29053337	USA	AECOPD	187,647	BMI > 30	In-hospital mortality	Improve
28979114	China Taiwan	Stable COPD	1,096	BMI > 24 or 27	The frequency of exacerbations	Improve
32752892	Dutch	AECOPD	604	BMI >25 or 30	The frequency of exacerbations	Improve
35255901	Korea	Stable COPD	1,264	BMI < 25 with chronic bronchitis	The frequency of exacerbations	Worsen
10588597	Denmark	Stable COPD	2,132	BMI < 20	Mortality from COPD	Worsen
25882802	Dutch	Stable COPD	505	Abdominal obesity by A/G%FM	Physical function	Improve
31504124	USA	Stable smokers	1,723	BMI > 25 or 30	Mortality	Improve
27568229	USA	Stable COPD	3,631	BMI > 30 or 35	QOL, 6MWD and severe AE	Worsen
26683222	USA	Stable COPD	1,096	BMI > 30	6MWD and SPPB	Worsen
30584475	Canada	Normal non-smokers	113,622	BMI > 25, 30, or 40	Odds of COPD	Worsen
35536690	USA	Stable COPD	98	DXA-assessed fat mass	Exercise capacity and physical activity	Worsen
25573407	USA	Stable COPD	84	BMI > 30	Responses to indoor PM exposure	Worsen
35674653	Korea	Normal women	1,644,635	BMI > 25 or 30 WC > 95	Incidence of COPD and asthma	Worsen
28280320	Korea	Mild COPD	618	BMI > 25	FVC	Worsen

COPD, chronic obstructive pulmonary disease; AE, acute exacerbation; BMI, body mass index; A/G%FM, android/gynoid percentage fat mass; QOL, quality of life; 6MWD, 6-min walk distance; SPPB, short physical performance battery; DXA, dual x-ray absorptiometry; PM, particle matter; FVC, forced vital capacity.

To enrich the clinical data of Chinese people on obesity and COPD, 120 patients hospitalized for AECOPD were enrolled in our study and divided into three groups of underweight, normal weight, and overweight based on BMI. As shown in **Table 2**, some lung function indices (FEV_1_% and FEV_1_/FVC) of overweight group were statistically superior to those of normal weight and overweight group, suggesting that proper weight gain might help slow the rate of decline in the lung function. In addition, diffusing capacity of the lungs for carbon monoxide and COPD assessment test also showed a positive trend in the overweight group. Despite the lack of statistical significance, overweight group had lower percentage of re-AE in 3 months and 1 year, indicting the better prognosis.

**Table 2:** Clinical characteristics of patients with COPD with different BMI.

Clinical Characteristics		BMI ≤ 18 (n = 14)	18 < BMI < 24 (n = 66)	BMI ≥ 24 (n = 40)	*P*-value
**Spirometry in stable period**	Age (year old)	71.5 ± 10.5	76.2 ± 9.0	74.1 ± 10.6	0.228
	Sex (M/F)	13/1	57/9	33/7	0.622
	FEV_1_ (L)	1.14 ± 0.26	**0.91 ± 0.31**	**1.15 ± 0.38**	**0.002**
	FEV_1_%	41.4 ± 8.8	**36.9 ± 12.5**	**45.8 ± 14.6**	**0.006**
	FVC (L)	2.45 ± 0.47	2.01 ± 0.68	2.15 ± 0.60	0.096
	FVC%	68.4 ± 10.4	60.5 ± 17.6	64.7 ± 13.0	0.201
	FEV_1_/FVC	**46.1 ± 5.7**	**46.4 ± 9.2**	**53.8 ± 10.9**	**0.001**
	DLCO [ml/(mmHg·min)]	6.43 ± 2.96	6.14 ± 3.54	9.22 ± 6.8	0.283
**Stable assessment**	CAT	22.7 ± 5.9	20.6 ± 6.6	19.0 ± 6.4	0.263
	mMRC	2.42 ± 0.67	2.40 ± 0.74	2.41 ± 0.75	0.995
**AE numbers in previous year**	0	1	3	2	0.682
	1–2	11	43	27	
	≥3	0	9	5	
**Laboratory examinations in AE period**	Eos (× 10^9^/L)	186 ± 194	112 ± 142	114 ± 98	0.175
	ESR (mm/L)	16.7 ± 10.2	21.2 ± 14.3	24.3 ± 16.9	0.31
	ALB (g/L)	34 ± 4	36 ± 4	37 ± 5	0.309
	CRP (mg/L)	30.2 ± 51.7	47.8 ± 68.1	42.7 ± 58.1	0.647
	PaO_2_ (mmHg)	83.6 ± 25.1	83.1 ± 29.3	82.3 ± 28.6	0.988
	PaCO_2_ (mmHg)	44.3 ± 13.8	49.8 ± 11.8	46.5 ± 10.0	0.213
**Prognosis**	Length of stay	12.6 ± 6.2	12.8 ± 3.4	12.6 ± 9.1	0.978
	In-hospital death	0 (0%)	3 (4.5%)	0 (0%)	0.284
	Re-AE in 1 month	1 (7.1%)	4 (6.7%)	4 (10.3%)	0.806
	Re-AE in 3 months	3 (21.4%)	11 (18.3%)	4 (10.3%)	0.469
	Re-AE in 6 months	4 (28.6%)	13 (21.7%)	9 (23.1%)	0.858
	Re-AE in 1 year	7 (50%)	23 (40.4%)	12 (32.4%)	0.49
	Death in 1 year	0 (0%)	0 (0%)	0 (0%)	1

COPD, chronic obstructive pulmonary disease; BMI, body mass index; FEV_1_, forced expiratory volume in one second; FVC, forced vital capacity; DLCO, diffusing capacity of the lungs for carbon monoxide; CAT, COPD assessment test; mMRC, modified British medical research council; AE, acute exacerbation; Eos, eosinophils; ESR, erythrocyte sedimentation rate; ALB, albumin; CRP, C-reactive protein. Bold means to highlight the statistically significant parameters.

### DEGs were discovered in COPD and obesity datasets

**Figure 1:**
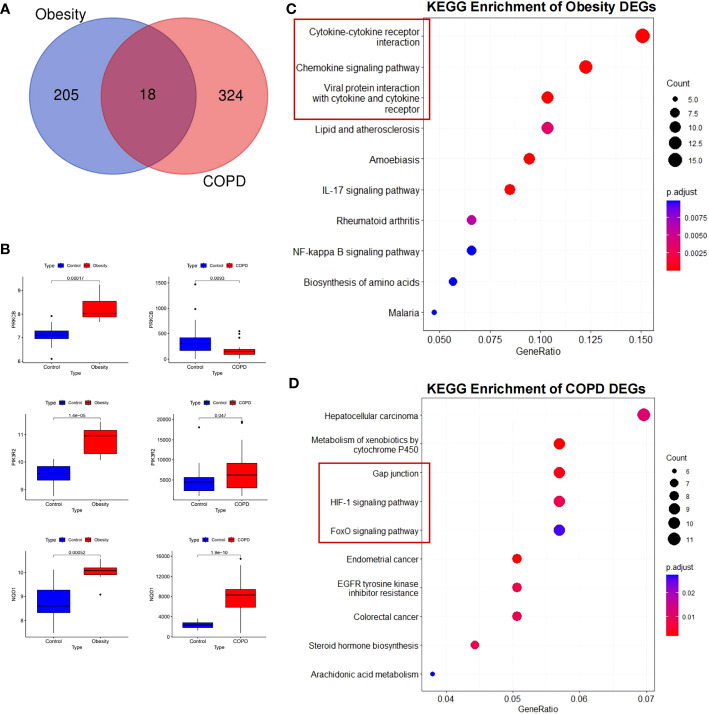
Differentially expressed genes and KEGG enrichment of obesity and COPD. **(A)** Differential genes in the three datasets. **(B)** Validation of differential expression of three crucial genes in GEO public datasets. Increased expression of PRKCB, PIK3R2, and NQO1 in obesity; increased expression of PIK3R2 and NQO1, decreased expression of PRKCB in COPD. **(C)** KEGG enrichment of obesity DEGs. **(D)** KEGG enrichment of COPD DEGs. KEGG, Kyoto Encyclopedia of Genes and Genomes; COPD, chronic obstructive pulmonary disease; PRKCB, protein kinase C beta; PIK3R2, phosphoinositide-3-kinase regulatory subunit 2; NOQ1, NADPH quinone oxidoreductase 1; DEGs, differentially expressed genes.

To explore the inherent pattern of gene expression and potential association between COPD and obesity, we analyzed DEGs in GEO public datasets. After filtering the duplicate and non-sense genes, a total of 342 DEGs in COPD and 223 DEGs in obesity were identified, including 18 overlapping DEGs (**Figure 1A**). Volcano maps of the DEGs are plotted in **Supplementary Figure 1A** and **Supplementary Figure 1B**. The inflammatory factors [C-X-C Motif Chemokine Ligand 8 (CXCL8), CXCL2, and matrix metalloproteinase 9 (MMP9)] were significantly increased and the lipid metabolism regulators (Apolipoprotein B (APOB) and Cholesteryl ester transfer protein (CETP)) were significantly decreased in patients with obesity. As for patients with COPD, the Activin A Receptor Type 2B (ACVR2B) and Transforming Growth Factor Beta 1 (TGFβ1), which were the key modulators of BMPR2 pathway, were significantly downregulated, as well as the obesity-related gene Apelin Receptor Early Endogenous Ligand (APELA). However, the BMP4 was remarkably upregulated. The identified 18 overlapping DEGs in obesity and COPD were referred to **Supplementary Tables 2, 3**. Of these genes, we found that the expression level of protein kinase C beta (PRKCB) was high in obesity but low in COPD. Expression of phosphoinositide-3-kinase regulatory subunit 2 (PIK3R2) and Reduced nicotinamide adenine dinucleotide phosphate (NADPH) quinone oxidoreductase 1 (NOQ1) genes was increased in obesity and COPD, respectively, compared with the controls (**Figure 1B**).

### KEGG, GSEA, and PPI analysis emphasized adipocytokines regulating inflammation and injury repairment

KEGG annotation analysis of DEGs in obesity revealed that the pathway mainly included cytokine-cytokine receptor interaction (P = 4.17E-05), viral protein interaction with cytokine and cytokine receptor (P = 9.32E-06), chemokine signaling pathway (P = 4.17E-05), and lipid and atherosclerosis (P = 0.0029) (**Figure 1C**). As for COPD, KEGG pathway analyses of DEGs were significantly enriched for gap junction (P = 0.003734), metabolism of xenobiotics by cytochrome P450 (P = 0.002103), Hypoxia-inducible factor 1-alpha (HIF-1) signaling pathway (P = 0.008497), and Forkhead box O (FoxO) signaling pathway (P =0.02568) (**Figure 1D**). Similarly, through GSEA, we confirmed that the cytokine and cytokine receptor genes were simultaneously upregulated in COPD and obesity but exhibited an opposite trend in the two pathological statuses (**Supplementary Figures 1C, D**).

To determine the interactions among the DEGs obtained above, we also generated PPI network by STRING analysis. It discovered that adipocytokines and proteases such as CXCL8, MMP9, and C-C Motif Chemokine Receptor 5 (CCR5) were hub genes in white adipose tissue of patients with obesity. Moreover, cell-junction proteins and growth factors such as Catenin beta-1 (CTNNB1), Epidermal growth factor (EGF), Epidermal growth factor receptor (EGFR), and Brain-derived neurotrophic factor (BDNF) were the hub genes in airway epithelial cells in COPD. Our results indicated that inflammation and injury repairment were the main and shared inherent patterns in obesity and COPD.

### WGCNA separately revealed the key modules of obesity and COPD

**Figure 2:**
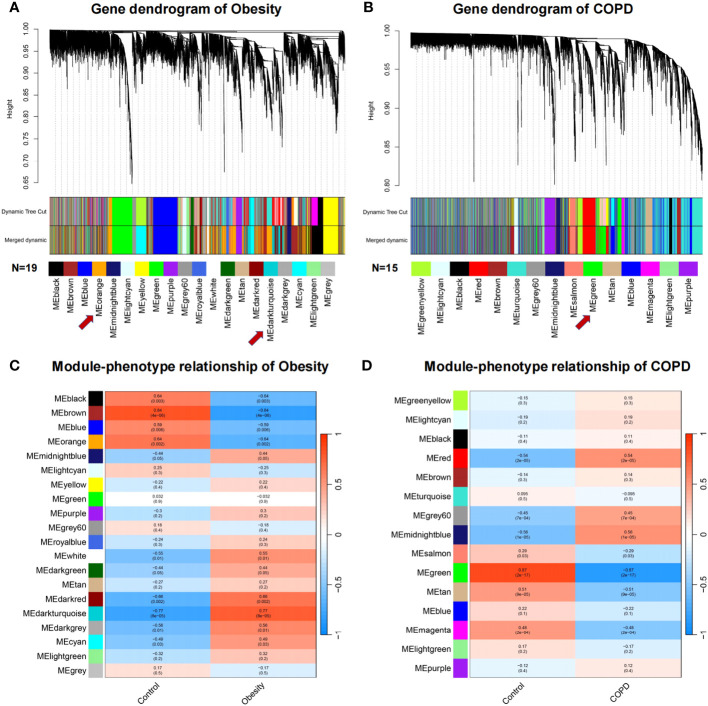
Weighted gene co-expression network analysis of obesity and COPD. **(A, B)** Network heatmap plot in the co-expression modules of obesity and COPD. Each leaf and branch on the tree represent a gene and a co-expression module. Each color below represents a co-expression gene module. **(C, D)** Heatmap between the correlation between modules and obesity, modules and COPD, respectively. Each cell contained the correlation coefficient and corresponding P-value. COPD, chronic obstructive pulmonary disease.

To describe the co-expression relationship of genes, WGCNA analysis was performed to identify the highly correlated gene modules closely related to obesity and COPD, respectively. The co-expression modules were showed by cluster dendrogram. Obesity and control groups revealed 19 gene modules, referred to leaf and branches in **Figure 2A**. The MEbrown was most significant correlated to obesity (correlation value, 0.84; P = 4E-06). The DEGs and hub gene were mainly distributed in MEorange (correlation value, −0.64; P = 0.002) and MEdarkturquoise (correlation value, 0.77; P = 8E-05) modules, and BMPR2 was distributed in MEmidnightblue module (correlation value, 0.44; P = 0.05) (**Figure 2C**). Fifteen gene modules could be enriched in the COPD and control groups (**Figure 2B**) among which MEtan showed the most significant correlation (correlation value, −0.51; P = 9E-05). The DEGs and Hub gene were mainly distributed in MEgreen (correlation value, −0.87; P = 2E-17), and BMPR2 was located in ME turquoise (correlation value, 0.095; P = 0.5) (**Figure 2D**).

### Adipocyte-specific knockout of BMPR2 caused lung injury and emphysema

BMPR2 is pivotal in maintaining pulmonary vascular homeostasis and the development of PAH, and BMPs are its ligands. As one of the 18 overlapping DEGs (shown in **Supplementary Tables 2, 3**) in obesity and COPD, PRKCB (encoded PKCβprotein) had the PPI with BMPR2. Secreted Phosphoprotein 1 (SPP1) (encoded osteopontin) also had interactions with BMPs. In addition, from the results of PPI network of obesity and COPD (**Supplementary Figures 2A**, **B**), the hub genes in COPD-PPI had close relationship with BMPR2. Therefore, BMPR2 might play a vital role in COPD-related lung injury and emphysema.

**Figure 3:**
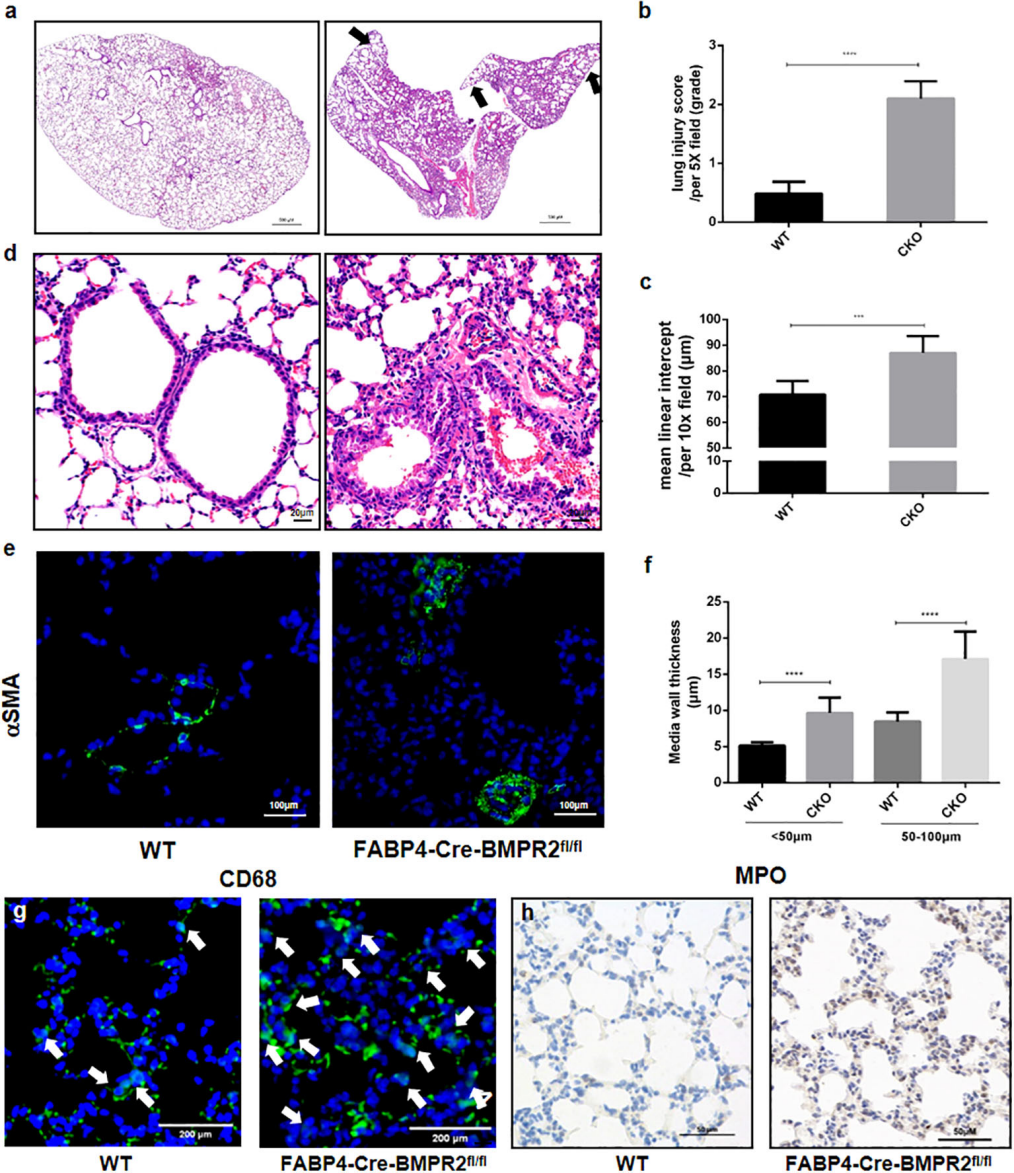
FABP4-Cre-BMPR2^fl/fl^ mice were susceptible to spontaneous lung inflammation, emphysema and vascular remodeling. **(A)** FABP4-Cre-BMPR2^fl/fl^ (CKO) mice had excessive inflammation in the whole lung and peripheral emphysema (black arrows, at 1x magnification). **(B, C)** Lung injury score and mean linear intercept (MLI) were calculated by the mean of 3-4 fields per slice and 5-6 slices per mouse. **(D)** CKO mice showed thicken vascular walls (at 20x magnification). **(E)** Immunofluorescent stain of α-SMA in the small lung arteries. **(F)** Media wall thickness of small vessels with different diameters was calculated by the mean of 2-3 fields per slice and 5-7 slices per mouse. **(G, H)** Infiltration of macrophages (CD68) and neutrophils (MPO) in the alveolus. ***P<0.001; ****P<0.0001. FABP4, fatty acid–binding protein 4; BMPR2, bone morphogenetic protein type II receptor; WT, wild type; CKO, conditional knockout; MPO, myeloperoxidase.

We demonstrated that FABP4-Cre-BMPR2^fl/fl^ (CKO) mice presented with spontaneous lung inflammation and significantly increased lung injury score, in comparison with WT mice (**Figures 3A, B**). Peripheral emphysema was also observed in CKO mice, with elevated mean linear intercept in the lung periphery (**Figures 3A, C**). In addition, **Figures 3D–F** showed that walls of small vessels became moderately thickened, along with the hypertrophy of pulmonary artery smooth muscle cells (PASMCs). As for inflammatory cells, we observed that CD68-marked macrophages and MPO-marked neutrophils were evidently upregulated in the alveolus of CKO mice (**Figures 3G, H**). Interestingly, lung inflammation, emphysema, and vascular remodeling are also pathological traits of COPD. We hypothesized that exacerbated inflammation and small artery remodeling synergistically resulted in right heart dysfunction, yet mice under 3 weeks old lacked mature compensatory mechanism and were prone to death.

**Figure 4:**
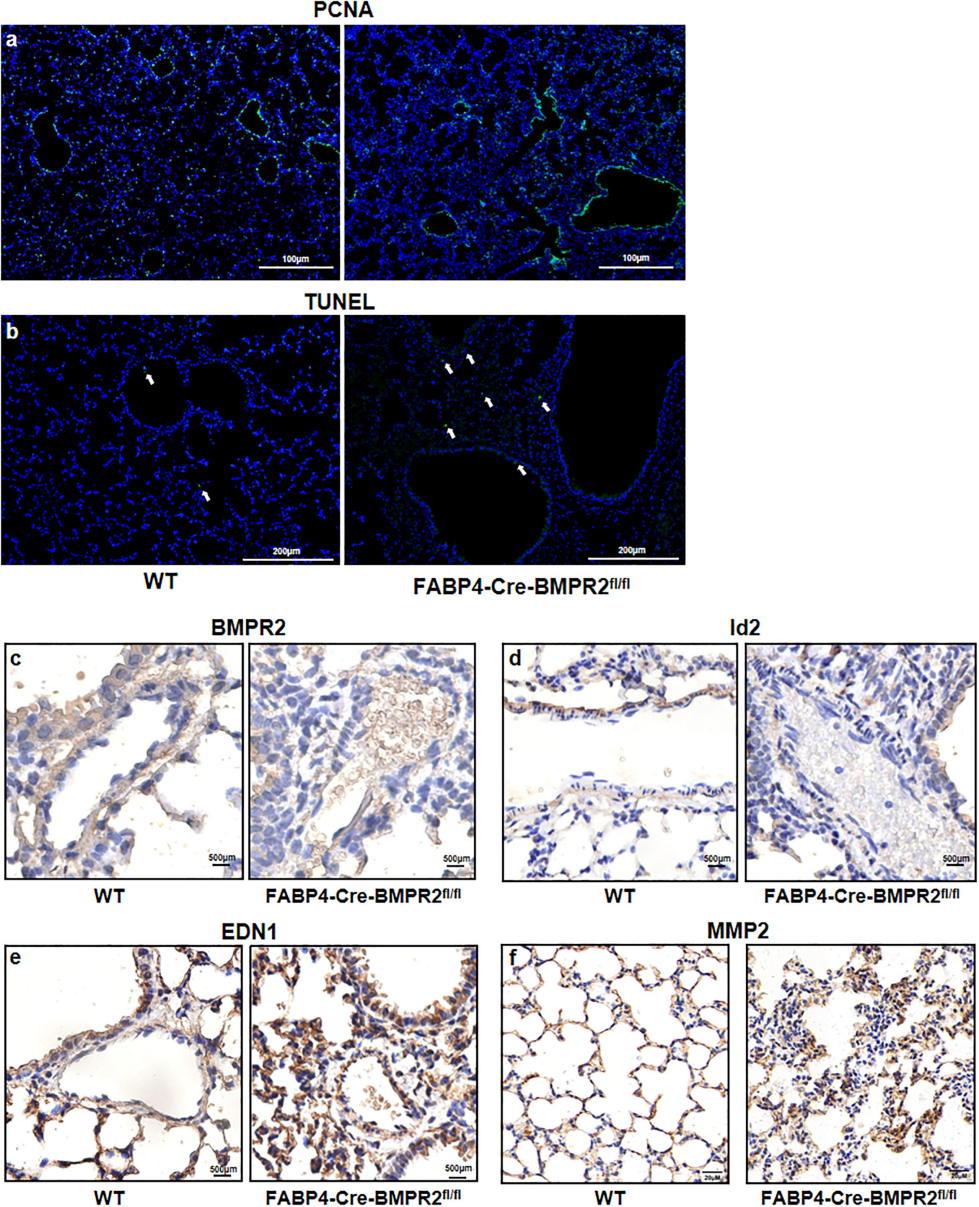
Preliminary exploration of the mechanisms of pathological changes in the lungs of FABP4-Cre-BMPR2^fl/fl^ mice. **(A)** Immunofluorescent stain of PCNA was not evident in the small lung arteries and not different in the whole lung between two groups. **(B)** TUNEL stain showed slightly increased numbers of apoptotic cells (white arrows) in the lungs of CKO mice. **(C, D)** Immunohistochemistry of BMPR2 and Id2 in the lung arteries. **(E)** Immunohistochemistry of EDN1 in the small airways and arterioles. **(F)** Immunohistochemistry of MMP2 in the alveolus. PCNA, proliferating cell nuclear antigen; TUNEL, terminal deoxynucleoitidyl transferase–mediated nick end labeling; FABP4, fatty acid–binding protein 4; BMPR2, bone morphogenetic protein type II receptor; Id2, inhibitor of DNA binding 2; EDN1, endothelin; MMP2, matrix metalloproteinase 2.

To investigate further mechanisms, we compared the difference of proliferation and apoptosis of lung’ cells between the WT and CKO groups, by performing immunological stain of proliferating cell nuclear antigen (PCNA) and TUNEL stain for the lung slices. PCNA was mainly expressed in the airways and did not differ in two groups (**Figure 4A**). Slightly increased number of apoptotic cells was observed in the CKO group (**Figure 4B**). Interestingly, BMPR2 and its targeted protein Id2 were declined in small arteries of CKO mice but not in small airways (**Figures 4C, D**). It demonstrated that the deficiency of BMPR2 in adipose tissue could influence its expression in other organs, especially in pulmonary vasculature. In addition, pro-contraction endothelin was upregulated in the small airways and arterioles of CKO mice (**Figure 4E**), but endothelin-converting enzyme 1 was similar in two groups (data not shown). Consistent with inflammatory cells, MMP2 was also increased in the alveolus of CKO mice (**Figure 4F**).

### Adipocytokines are involved the pathogenesis of lung injury in BMPR2-deficient adipocytes

**Figure 5:**
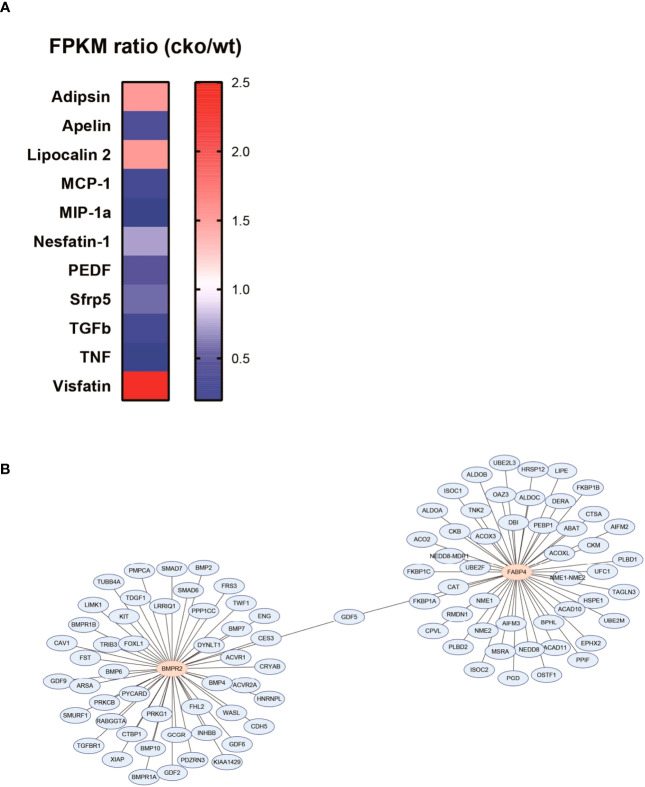
Adipocytokines involved the pathogenesis of lung injury in BMPR2-deficient adipocytes. **(A)** Differently expressed adipocytokines in the adipocytes were calculated by the FPKM ratio of CKO and WT group. **(B)** PPI network of BMPR2-related proteins and FABP4-related proteins. FPKM, fragments per kilobase of exon model per million mapped fragments; WT, wild type; CKO, conditional knockout; PPI, protein–protein interaction; FABP4, fatty acid–binding protein 4; BMPR2, bone morphogenetic protein type II receptor.

To preliminarily explore the roles of BMPR2-defiecient adipocytes, we re-analyzed the RNA sequence data of adipocytes in WT and CKO mice, which was uploaded by Qian et al. (Sequence Read Archive (SRA) accession: PRJNA611934) (23). Adipocytes of each group were pooled from four individual mice’s inguinal adipose tissue. We compared the differential expression of multiple adipocytokines and listed those with fragments per kilobase of exon model per million mapped fragments >1 and fold change >1.5 (**Figure 5A**). We observed the dramatically decreased apelin and slightly increased adipsin in the CKO group (**Figure 5A**). Moreover, we searched the top 50 BMPR2-associated proteins and FABP4-associated proteins and identified as the sole conjunct gene (**Figure 5B**). Thus, we postulated that apelin, adipsin, and growth differentiation factor 5 (GDF5) might participate in the pathological process that BMPR2-deficient adipocytes influenced lung structure.

## Discussion

In our study, we discovered that patients with obesity with COPD had improved prognosis and lung function. In addition, several potential inflammatory hub genes (CXCL8, MMP9, and CCR5) and adipocytokine hub genes (PRKCB, PIK3R2, NOQ1, BMP4, ACVR2B, and TGFβ1) were identified. Further KEGG analysis, GSEA, PPI network, and WGCNA showed that inflammation and injury repairment were the main and shared inherent patterns in obesity and COPD. We also found the adipocyte-specific BMPR2 knockout mice to spontaneously develop lung injury and emphysema, in which adipsin and GDF5 might be involved in the pathogenesis. Our study indicated that adipocytes dysfunction might promote lung injury, which could provide therapeutic targets and biomarkers for patients with COPD.

Our cohort analysis of patients with COPD showed that the group with BMI ≥24 had decreased percentage of re-AE in 3 months and 1 year, indicating a favorable prognosis. Similar findings were also shown in the previous studies (32–34). BMI is the most frequently used anthropometric tool to measure the fatness. Although it is convenient to access, BMI is a very crude measurement of obesity (35). It could not distinguish between fat mass and free-fat mass (36). BMI is hard to identify the fat distribution like the abdominal fat or gluteo-femoral fat (37). Further studies are needed to explore the relationship between different body components and prognosis of COPD.

**Table 1** demonstrates the inconsistent effects of obesity on COPD. However, multiple confounding factors between COPD and obesity influence the prognosis of patients. For example, patients with overweight and obesity were more likely to be misdiagnosed with COPD and be prescribed with inhaled medications due to dyspnea (38). In addition, patients with obesity often had complications that led to dyspnea and hypoxemia, such as functional restriction, congenital heart failure, and obstructive sleep apnea (39).

In recent years, bioinformatic tools have been widely used for identifying DEGs and novel markers in COPD (40–42). In our bioinformatic analysis, we found several DEGs including inflammatory factors (CXCL8, CXCL2, and MMP9) and lipid metabolism regulators (APOB and CETP), which were also reported in some previous studies of COPD (43–45). Similarly, some DEGs of the COPD dataset, like the regulator of BMPR2 pathway and APELA, are also the obesity-related genes. NOQ1, one of antioxidant response elements (46), was significantly increased in both obesity and COPD, which is consistent with the previous results (41). We also observed an upregulation of PIK3R2 in COPD and obesity. Inhibition of PIK3R2 could increase the levels of tight junction protein and protect the function of epithelial cells (47). The expression levels of PRKCB differed in obese and COPD datasets. PRKCB plays multiple roles in regulating cell survival and apoptosis (48), indicating that it might determine different cellular fate in adipocytes and bronchial epithelial cells. GSEA and KEGG analysis of obesity showed an enhancement of cytokine and chemokine pathways, whereas the results of COPD showed an upregulation of repair-related genes such as cell junctions, HIF1α, and FOXO3. On the basis of the above results, we speculate white adipose tissue to be a potential endocrine organ that influences lung inflammation and injury repair, by secreting systemic cytokines and chemokines.

Mutation and deficiency of BMPR2 leads to an obvious perivascular inflammation and muscularization in pulmonary hypertension (49). To date, there are limited studies on BMPR2 and COPD. BMPR2 is significantly decreased in lung tissue of patients with COPD (50). Moreover, BMPR2 mutation could increase the susceptibility of COPD (51). Hence, deficiency of BMPR2 may aggravate the progression of COPD. Our study utilized adipocyte-specific BMPR2 knockout mice and found that CKO mice presented with a COPD phenotype, with emphysema, lung injury, and arteriole remodeling. On the basis of the previous study by Qian et al., it is possibly due to elevated level of serum TNFα, leading to increased infiltration of inflammatory cells into lung and alveolar destruction (23). Our results do not show the disbalance of proliferation and apoptosis to be a major cause of lung structural damage.

Adipocytokines are bioactive mediators secreted by adipocytes, and we screened out three potential adipocytokines in our study. Apelin was decreased in the white adipose tissues of the CKO group. Consistently, APELA was downregulated in small airways of patients with COPD. Similar results have been reported to decrease apelin in patients with pulmonary artery hypertension secondary to COPD (52). Apelin plays protective role in obesity, by regulating the inflammation and oxidative stress (53). Moreover, adipsin was increased in white adipose tissues of the CKO group. It was regarded as a novel serum biomarker of COPD-associated pulmonary hypertension (54). Some adipocytokines like adipsin were similarly elevated in patients with chronic bronchitis (55). GDF5 was predicted by PPI network as a core protein between BMPR2- and FABP4-interating network. GDF5 was also known as BMP14, belonging to family of BMPs. Its function is still unknown in COPD, but administration of GDF5 could promote cartilage repair by inhibition of inflammation in osteoarthritis models (56). Moreover, FABP4-GDF5 transgenic mice showed increased sensitivity to insulin on a high-diet food, probably by promoting thermogenesis of white adipose tissue (57).

The number of clinical cases in our study being relatively low is a major limitation. Further studies on body composition and nutrition of COPD are needed. Moreover, the inclusion criterion was hospitalized patients with AECOPD. Because Shanghai Zhongshan Hospital is a grade-A tertiary hospital, participants might have several comorbidities, such as hypertension, diabetes, and coronary heart disease. Larger, multi-center studies will be conducted in the future. Because of premature death of FABP4-Cre-BMPR2^fl/fl^ mice after weaning, it was difficult to measure the pulmonary function parameters. We plan to breed FABP4-CreERT2-BMPR2^fl/fl^ mice and administered tamoxifen via intraperitoneal injection or gavage in adulthood to induce the knockout of BMPR2 in adipocytes. We are also planning to confirm whether TNFα or apelin is the main culprit of lung injury in our future research. Co-culture of mouse pulmonary endothelial cells and BMPR2-knockdown adipocytes will be performed and supplemented with anti–TNFα-neutralizing antibody or apelin receptor agonist. Mouse adipocytes were differentiated from 3T3-L1 cell line. More experiments are needed to be designed and conducted to further validate the mechanism of protective role of GDF5 in obesity and COPD.

## Conclusion

In summary, inflammation and abnormal repair might be potential mechanisms of the pathological association between obesity and COPD. Moreover, this study innovatively supplemented a pathogenic link between adipocyte dysfunction and lung injury, indicating adipocytes potentially to be a new class of key cells in the development of COPD. This provides new insight into the pathological mechanism and a promising therapy of COPD.

## Data availability statement

The original contributions presented in the study are included in the article/**Supplementary Material**. Further inquiries can be directed to the corresponding authors.

## Ethics statement

The studies involving humans were approved by Institutional Ethics Committee of Shanghai Zhongshan Hospital. The studies were conducted in accordance with the local legislation and institutional requirements. The participants provided their written informed consent to participate in this study. The animal study was approved by Institutional Ethics Committee of Shanghai Zhongshan Hospital. The study was conducted in accordance with the local legislation and institutional requirements. The clinical study of patients with AECOPD has been approved by Institutional Ethics Committee of Shanghai Zhongshan Hospital (No. B2015-119R). Informed written consent for clinical data collection and anonymous publication has been acquired from each patient on admission.

## Author contributions

Conception and design: S-JZ and W-PH; experiment: S-JZ, B-FH, and W-PH; analysis and interpretation: X-ZQ and JZho; clinical study design: W-PH and JZha; drafting the manuscript: S-JZ, SS, and W-PH. All authors read and approved the final manuscript.
