# Supplementary material for: Adipocyte dysfunction promotes lung inflammation and aberrant repair: a potential target of COPD
Source: Front Endocrinol (Lausanne). 2023 Oct 10;14:1204744. doi: 10.3389/fendo.2023.1204744 (PMC10597776; doi:10.3389/fendo.2023.1204744)
Supplement: Supplementary file 1 [file DataSheet_1.docx]

Adipocyte Dysfunction Promotes Lung Inflammation and Aberrant Repair: A Potential Target of COPD

**Si-jin Zhang^1†^ , Xian-zheng Qin^2†^, Jie Zhou^3†^, Bin-feng He^4^, S****urendra Shrestha^5^, Jing Zhang^4*^, Wei-ping Hu^4*^**

^1^ Department of Pulmonary and Critical Care Medicine,Ruijin Hospital, Shanghai Jiao Tong University School of Medicine, Shanghai, 200025, China

^2^ Department of Gastroenterology,Ruijin Hospital, Shanghai Jiao Tong University School of Medicine, Shanghai, 200025, China

^3^ Department of Hematology, Tongji Hospital of Tongji University, Tongji University School of Medicine, Tongji University, Shanghai, China

^4^ Department of Pulmonary and Critical Care Medicine, Zhongshan Hospital, Fudan University, Shanghai 200032, China

^5^ Emergency Department, Om Aasha Hospital Pvt. Ltd. Dhanghadi, Nepal

***Correspondence**

Wei-ping Hu

Department of Pulmonary and Critical Care Medicine, Zhongshan Hospital, Shanghai Medical College, Fudan University, Shanghai, China, 200032

Email: [wphu@foxmail.com](mailto:wphu@foxmail.com)

Jing Zhang

Department of Pulmonary and Critical Care Medicine, Zhongshan Hospital, Shanghai Medical College, Fudan University, Shanghai, China, 200032

Email: [zhang.jing@zs-hospital.sh.cn](mailto:zhang.jing@zs-hospital.sh.cn)

**Supplementary Figures**

## Supplementary Figure 1


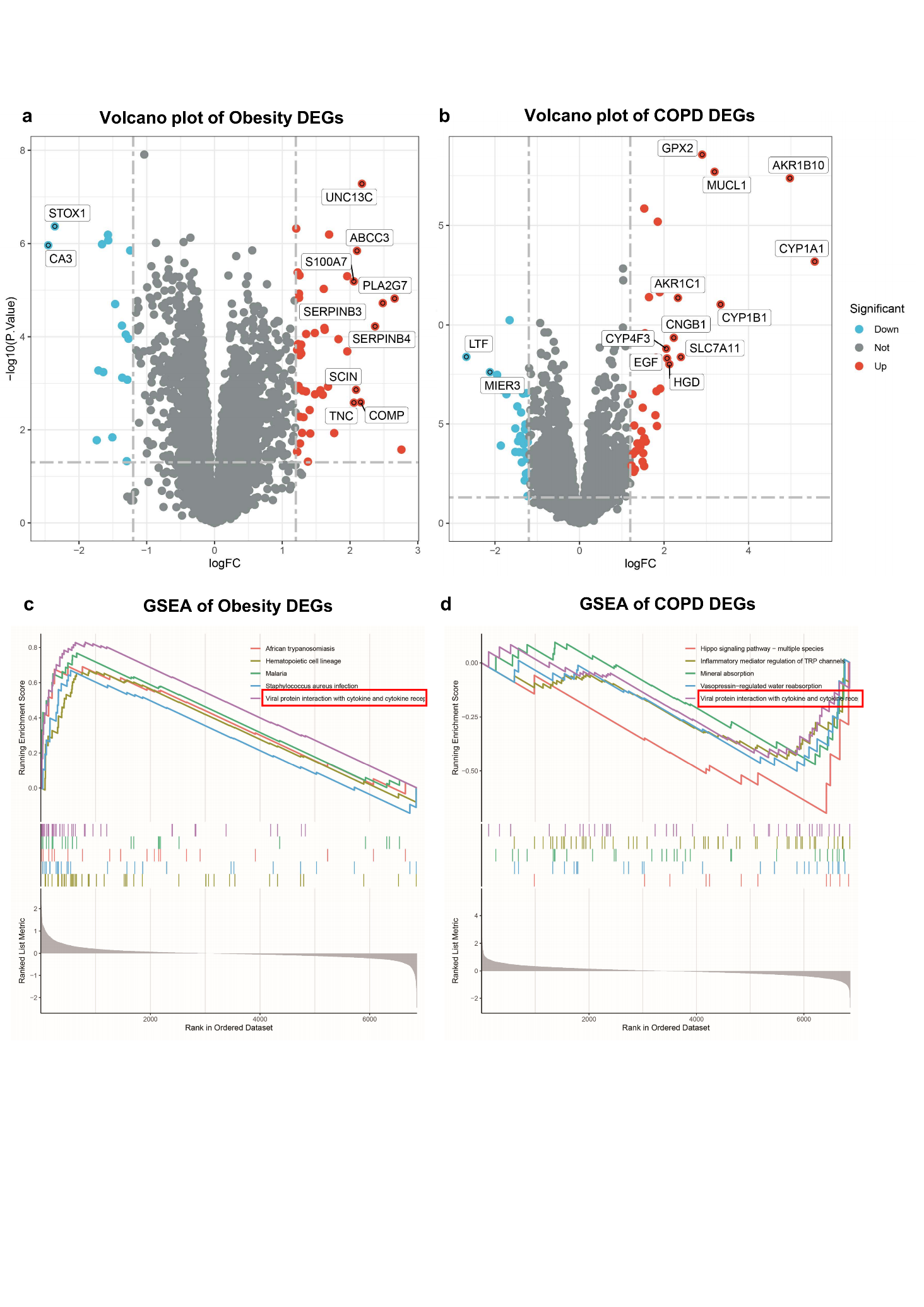


**Supplementary Figure 1.** **Volcano plot and GSEA plot of the DEGs of obesity and COPD**

**(a&b)** The volcano plots of Obesity DEGs and COPD DEGs. Each red dot represents upregulated gene and each blue dot represents downregulated gene; **(c&d)** GSEA of obesity and COPD DEGs. The hub genes are predominant in the related pathways; Abbreviations: GSEA, gene set enrichment analysis; DEGs, differentially expressed genes; COPD, chronic obstructive pulmonary disease.

## Supplementary Figure 2


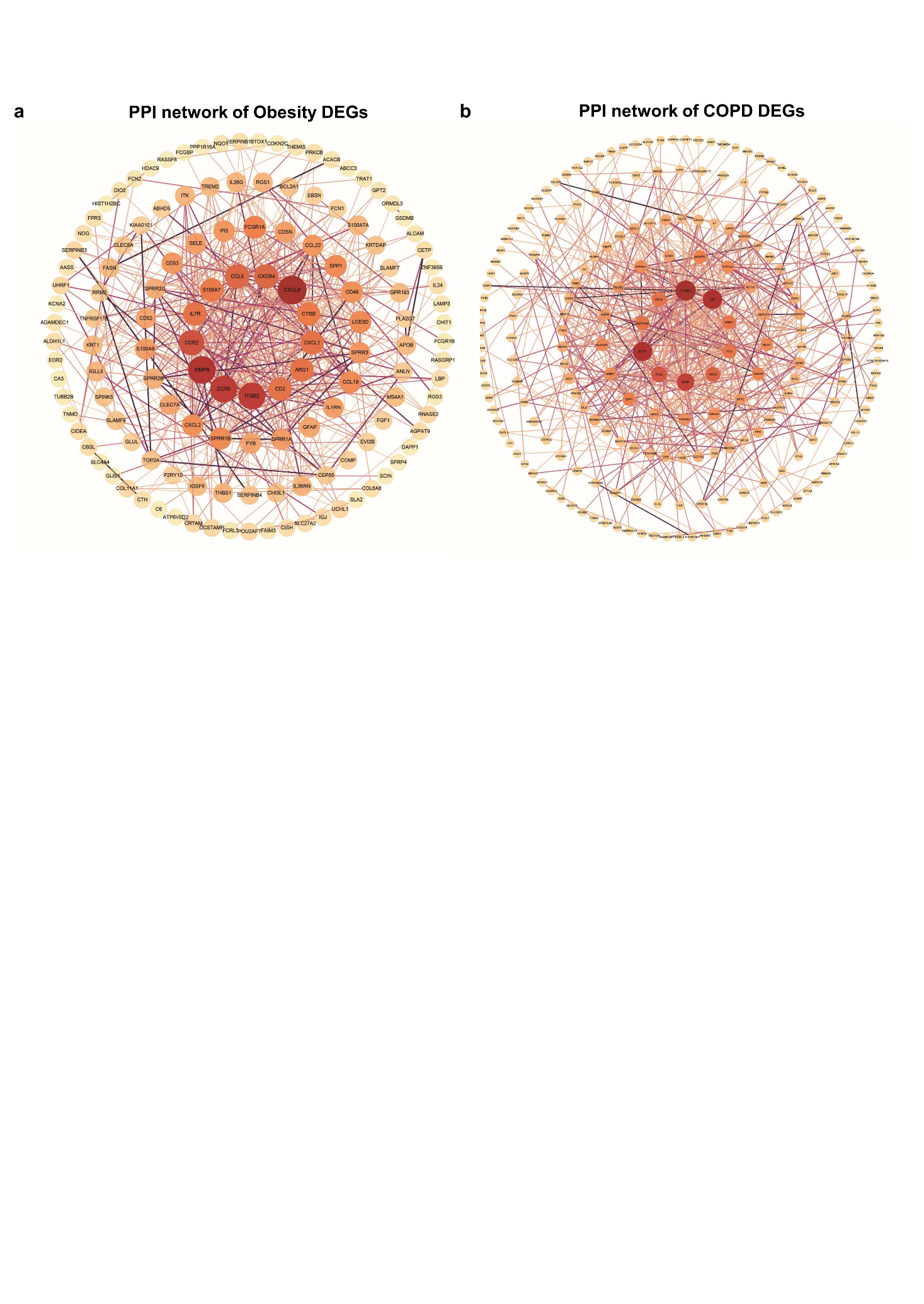


**Supplementary Figure 2.** **PPI network and hub genes of the DEGs of obesity and COPD**

**(a&b)** Construction of PPI network maps with hub genes of obesity and COPD. Red nodes represent key genes. The darker the nodes the stronger correlation with the hub gens are. Abbreviations: PPI, protein-protein interaction; DEGs, differentially expressed genes; COPD, chronic obstructive pulmonary disease.

## Supplementary Tables

**Supplementary Table 1. Antibody information**

| **Target** | **Catalog.** | **Brand** | **Dilution** |
| --- | --- | --- | --- |
| αSMA | 19245 | CST | 1:500 |
| PCNA | 10205-2-AP | Proteintech | 1:500 |
| BMPR2 | A18079 | abclonal | 1:100 |
| ECE1 | A5638 | abclonal | 1:100 |
| EDN1 | A18004 | abclonal | 1:100 |
| Id2 | A0996 | abclonal | 1:100 |
| MMP2 | GB11130 | Servicebio | 1:100 |
| CD68 | orb10343 | biorybt | 1:200 |
| MPO | GB11224 | Servicebio | 1:100 |
| Alexa Fluor® 488 AffiniPure Goat Anti-Rabbit IgG (H+L) | GB25303 | Servicebio | 1:200 |

**Supplementary Table 2. Identification of 18 DEGs in COPD**

| **Gene Symbol** | **Gene Title** | **Log FC** | **Adj. P Value** |
| --- | --- | --- | --- |
| UCHL1 | Ubiquitin C-Terminal Hydrolase L1 | 4.76 | 1.60E-10 |
| TUBB2B | Tubulin Beta 2B Class IIb | 1.60 | 3.59E-03 |
| SPRR3 | Small Proline Rich Protein 3 | 2.24 | 9.59E-04 |
| SPRR1A | Small Proline Rich Protein 1A | 1.39 | 3.16E-02 |
| SPRR1B | Small Proline Rich Protein 1B | 1.47 | 5.28E-03 |
| SPP1 | Secreted Phosphoprotein 1 | 3.02 | 2.80E-07 |
| PLA2G7 | Phospholipase A2 Group 7 | 1.55 | 5.46E-03 |
| PIK3R2 | Phosphoinositide-3-Kinase Regulatory Subunit 2 | 1.29 | 3.56E-03 |
| NQO1 | NAD(P)H Quinone Dehydrogenase 1 | 2.03 | 1.32E-09 |
| LINC00942 | Long Intergenic Non-Protein Coding RNA 942 | 2.42 | 1.70E-04 |
| IGSF6 | Immunoglobulin Superfamily Member 6 | 1.22 | 3.41E-02 |
| IGLC1 | Immunoglobulin Lambda Constant 1 | -1.61 | 4.39E-02 |
| IGK | Immunoglobulin Kappa Locus | -1.69 | 1.65E-02 |
| GPAT3 | Glycerol-3-Phosphate Acyltransferase 3 | 1.41 | 4.78E-06 |
| ATP6V0D2 | ATPase H+ Transporting V0 Subunit D2 | 2.01 | 6.16E-04 |
| CLEC5A | C-type Lectin Domain Family 5 Member A | 1.88 | 4.16E-03 |
| ABCC3 | ATP Binding Cassette Subfamily C Member 3 | 1.29 | 6.08E-04 |
| PRKCB | Protein Kinase C Beta | -1.23 | 1.46E-02 |

**Supplementary Table 3. Identification of 18 DEGs in Obesity**

| **Gene Symbol** | **Gene Title** | **Log FC** | **Adj. P Value** |
| --- | --- | --- | --- |
| UCHL1 | Ubiquitin C-Terminal Hydrolase L1 | 1.83 | 4.46E-03 |
| TUBB2B | Tubulin Beta 2B Class IIb | 1.64 | 3.67E-03 |
| SPRR3 | Small Proline Rich Protein 3 | 2.29 | 2.49E-03 |
| SPRR1A | Small Proline Rich Protein 1A | 2.70 | 2.75E-03 |
| SPRR1B | Small Proline Rich Protein 1B | 2.55 | 2.00E-03 |
| SPP1 | Secreted Phosphoprotein 1 | 3.53 | 1.35E-03 |
| PLA2G7 | Phospholipase A2 Group 7 | 2.66 | 2.01E-03 |
| PIK3R2 | Phosphoinositide-3-Kinase Regulatory Subunit 2 | 1.24 | 1.25E-03 |
| NQO1 | NAD(P)H Quinone Dehydrogenase 1 | 1.25 | 5.56E-03 |
| LINC00942 | Long Intergenic Non-Protein Coding RNA 942 | 1.29 | 1.37E-02 |
| IGSF6 | Immunoglobulin Superfamily Member 6 | 1.21 | 6.25E-03 |
| IGLC1 | Immunoglobulin Lambda Constant 1 | 1.86 | 1.32E-02 |
| IGK | Immunoglobulin Kappa Locus | 1.51 | 2.46E-02 |
| GPAT3 | Glycerol-3-Phosphate Acyltransferase 3 | -2.03 | 1.03E-03 |
| ATP6V0D2 | ATPase H+ Transporting V0 Subunit D2 | 1.32 | 1.95E-02 |
| CLEC5A | C-type Lectin Domain Family 5 Member A | 1.23 | 8.92E-03 |
| ABCC3 | ATP Binding Cassette Subfamily C Member 3 | 2.12 | 6.97E-04 |
| PRKCB | Protein Kinase C Beta | 1.25 | 5.06E-03 |
